# Routine Management of Microalgae Using Autofluorescence from Chlorophyll

**DOI:** 10.3390/molecules24244441

**Published:** 2019-12-04

**Authors:** Toshiyuki Takahashi

**Affiliations:** Department of Chemical Science and Engineering, National Institute of Technology (KOSEN), Miyakonojo College, Miyakonojo, Miyazaki 885-8567, Japan; mttaka@cc.miyakonojo-nct.ac.jp; Tel.: +81-986-47-1219

**Keywords:** algae, chlorophyll fluorescence, automated cell counter, three-dimensional fluorescence excitation–emission matrix spectroscopy

## Abstract

From a high-potential biomass perspective, microalgae have recently attracted considerable attention due to their extensive application in many areas. Although studies searching for algal species with extensive application potential are ongoing, technical development for their assessment and maintenance of quality in culture are also critical and inescapable challenges. Considering the sensitivity of microalgae to environmental changes, management of algal quality is one of the top priorities for industrial applications. Helping substitute for conventional methods such as manual hemocytometry, turbidity, and spectrophotometry, this review presents an image-based, automated cell counter with a fluorescence filter to measure chlorophyll autofluorescence emitted by algae. Capturing chlorophyll-bearing cells selectively, the device accomplished precise qualification of algal numbers. The results for cell density using the device with fluorescence detection were almost identical to those obtained using hemocytometry. The automated functions of the device allow operators to reduce working hours, for not only cell density analysis but simultaneous multiparametric analysis such as cell size and algal status based on chlorophyll integrity. The automated device boldly supports further development of algal application and might contribute to opening up more avenues in the microalgal industry.

## 1. Introduction

As is well known, microalgae as primary producers are an important biomass that support freshwater and marine ecosystems. In addition to an established role in nature, they also provide a high-potential biomass for industrial applications including health and medical areas, food and nutrition science, aquaculture, environmental applications, and chemical production [1,2,3,4,5,6,7,8,9,10,11]. Regardless of the choice of algal species for an application, assessment and maintenance of their quality in culture are critical challenges [11,12]. It stands to reason that the quality assurance of microalgae, which are sensitive to environmental changes, is of particular importance [13,14]. Consequently, routine control, maintenance, and management of algal quality in culture is one of the top priorities in industrial applications.

In addition to describing general methods for assessing algal quality, this review presents a new method for easy and rapid evaluation of algal numbers and status using a compact, automated, image-based cell counter with a fluorescence filter for measuring chlorophyll autofluorescence. This method has the potential to relieve researchers and engineers of laborious and time-consuming routine tasks to check algal health and numbers. This will contribute to the further development of algal applications.

## 2. Standard Methods to Evaluate Cells

### 2.1. Conventional Methods to Evaluate Cells

#### 2.1.1. Microscopy and Hemocytometry 

**Figure 1:**
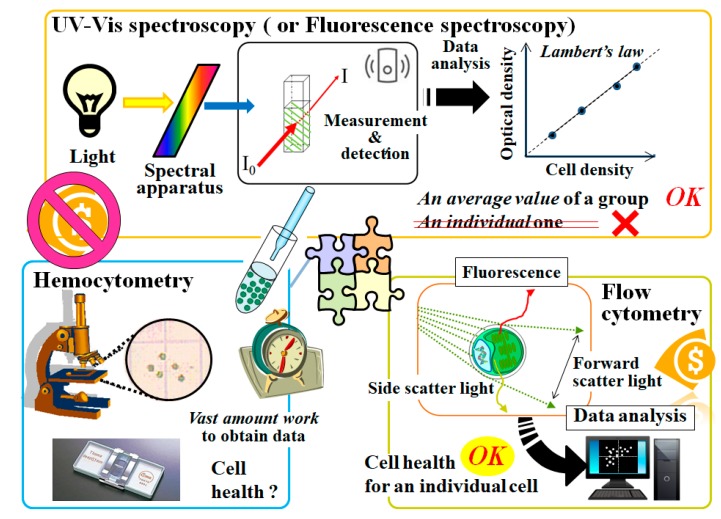
Several standard methods to evaluate microorganisms such as bacteria and microalgae. These include optical density measurements based on spectroscopy, hemocytometry, and flow cytometry.

As is the case with bacteria and mammalian cultured cells, microscopy and ultraviolet–visible (UV–Vis) spectroscopy are conventional tools in evaluating microalgae. Microscopy is adequately available for microalgae that are 2–10 μm in diameter. Actually, microscopy has elucidated characteristics of several microalgae. Hemocytometry using microscopy helps us determine a tally of the number of algae in culture manually (Figure 1). This method can be made available at a low cost. However, vast amounts of time for obtaining data have been expended for studies using these microscopic techniques. Use of these techniques could potentially render studies vulnerable to user bias and the misuse of hemocytometry [15]. With the determination of the cell density set aside, determining the cell status based on aesthetics alone is not an easy practice.

#### 2.1.2. Spectroscopy and a Method Based on a Chlorophyll Meter

Turbidity and optical density (OD) measurements have been conventionally used for the determination of cell density in microbiology [16,17]. The cell density can be determined from the analog of the Beer–Lambert law. This method, independent of molecular absorption, is also good for microalgae. 

In contrast to turbidity, some methods based on either UV–Vis spectroscopy or spectrofluorometry have dared to apply molecular absorbance or emission to detect chlorophyll in microalgae. The representative tool is a chlorophyll meter (Aquaread’s chlorophyll meter from AQUAREAD Ltd., Chlorophyll meter CHL-5Z from Kasahara chemical instruments corp., and others), which allows a non-destructive measurement as with turbidity. As is commonly known, chlorophyll is the green pigment found in photosynthetic organisms such as plants and phytoplankton, and is vital for photosynthesis. Measuring chlorophyll in water helps one assess the abundance of phytoplankton. UV–Vis spectroscopy and spectrofluorometry based on chlorophyll properties are also useful to evaluate algal health since chlorophyll integrity reflects the overall cell status of microalgae [11,12,13,14,18,19]. 

A spectrophotometer, as infrastructure equipment in a standard biological laboratory, method can be made available at a low cost. However, it is to be noted that the concentration of cells estimated by spectroscopy must be confirmed using a standard curve, unlike the use of other methods such as the hemocytometer and plate count. The value obtained via spectroscopy against cell mass is specific to each species of the microorganism [17]. In addition to less sensitivity at low cell densities than methods based on absorbance and emission, values obtained using OD measurement are also subject to influence of cell size change [20]. If chlorophyll integrity is deteriorated due to change in temperature, pH (e.g., heat and acidic environment), and other uncertain factors, the prepared standard curve might be rendered useless. In fact, pigments contents such as chlorophyll and carotenoids change as a result of algal acclimation to light condition in laboratory culture, outdoor photo-bioreactors, and nature [21,22]. Moreover, these methods evaluate more of an overall status of the culture than cell status of an individual cell. In other words, management of microalgae culture without visual observation may overlook the signs of microalgae with unhealthy chlorophyll. 

#### 2.1.3. Spectrofluorometry and Flow Cytometry

Spectrofluorometry and flow cytometry (FCM) are mostly used for detailed analyses [14,23,24,25,26,27] since they are generally higher-cost equipment than a microscope and UV–Vis spectrophotometer. These methods using fluorescence are vastly superior in both selectivity and sensitivity to spectroscopy, based on OD and absorbance (Figure 1). Moreover, fluorescence is also useful for sorting particles (or cells) of interest in heterogeneous populations. The cell density estimated by spectrofluorometry can be also determined from the analog of the Beer–Lambert law in the same manner as that by spectroscopy. 

Analogously with spectrofluorometry, FCM can detect chlorophyll autofluorescence of algae. Furthermore, unlike UV–Vis spectroscopy and spectrofluorometry, FCM can evaluate multiparametric properties of individual alga [28,29] (Figure 1). Recently, FCM has also been useful to ascertain total cell counts. FCM helps us evaluate cells in suspension more rapidly than microscopy-based hemocytometry. However, accurate evaluation of target cells requires sophisticated skills to set up measurement parameters at the outset of FCM. The salient difficulty is ascribable to the measurement principle that the technique does not capture cells of interest visually. 

#### 2.1.4. Dry Weight Measurement of Biomass

In analogy with other biomass, gravimetric determination of microalgae has been conventionally and frequently used for evaluation of microalgal biomass [20,30]. Although there is not any optical analysis and it might poorly inform us about culture health, the method using infrastructure equipment such as a microbalance can also be made available at a low cost. 

However, the method requires at least relatively large amounts (mg-order) of sample material size because of the measure of precision using a microbalance. In addition, the method requires the necessity to carefully wash the sample pellet for elimination of inorganic materials. Considering a high-throughput research, every small volume experiment such as screening assays in a microwell plate and applications requiring feedback control [20], dry weight measurement exhibits time-consuming and lower capability than any other optical analyses. 

### 2.2. A Recently Visualization-Based Automated Cell Counter to Evaluate Cells Such as Cultured Animal Cells

To reduce time expenditure of cell health checks using manual hemocytometry, commercially available and image-based automated cell counters (TC20^TM^ automated cell counter (Bio Rad Laboratories Inc., Hercules, CA, USA), Countess^TM^ II Automated Cell Counter (Thermofisher Scientific Inc., Waltham, MA, USA), Automated Cell Counter model R1 (Olympus Corp., Shinjuku-ku, Tokyo, Japan), FACSCOPE^TM^ B (Curiosis Inc., Gangnam-gu, Seoul, Korea), Corning^®^ Cell Counter (Corning Inc., Tewksbury, MA, USA), DigitalBio (NanoEnTek Inc., Guro-gu, Seoul, Korea), and others) have been introduced into cell culture experiments. Standard target cells for these instruments are cultured animal cells, e.g., human, rat, and mouse, which are generally larger than microalgae. In fact, these instruments with cell recognition algorithms help researchers not only shorten their routine tasks to check cell numbers but also guarantee their cell health through a viability assay by the assistance of specific stain solutions such as Trypan Blue. Almost all cell counters, except for Cell Counter model R1 from Olympus Corp., generally use a digitally monochrome camera as the optical principle rather than a lens. Although their attached cameras are barely able to detect cultured animal cells, they are thereby insufficient for microalgae detection due to low-resolution image production. Bright-field and low-resolution images obtained from standard cameras of each cell counter are troublesome for discrimination of small target cells, such as microalgae, from other particles such as debris, small stains and spots, and microbubbles in the counting chamber [11]. Most automated cell counters face a technical challenge in directly introducing that into cell management for small cells.

## 3. A New Method to Detect and Evaluate Microalgae Using Chlorophyll Autofluorescence

### 3.1. Advantages to Drawing on Chlorophyll Fluorescence from Chloroplast(s) on a Microalgal Management

**Figure 2:**
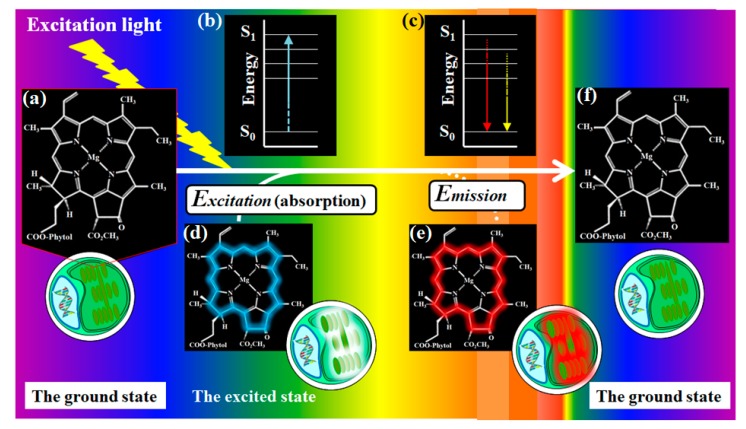
Autofluorescence emitted from chlorophyll molecules of a chloroplast in microalgae. Panels (**a**,**d**–**f**) are image-conscious figures of a chlorophyll molecule. Panels (**b**,**c**) are graphical interpretations. When the ground state of chlorophyll (a) is excited by an appropriate excitation light source, light energy from the light is absorbed by chlorophyll molecules (b,d). To return to a balanced state (the ground state) (f) from the excited state (d), the absorbed energy is eradiated as autofluorescence (c,e). Excited chlorophyll molecules emit red fluorescence.

**Figure 3:**
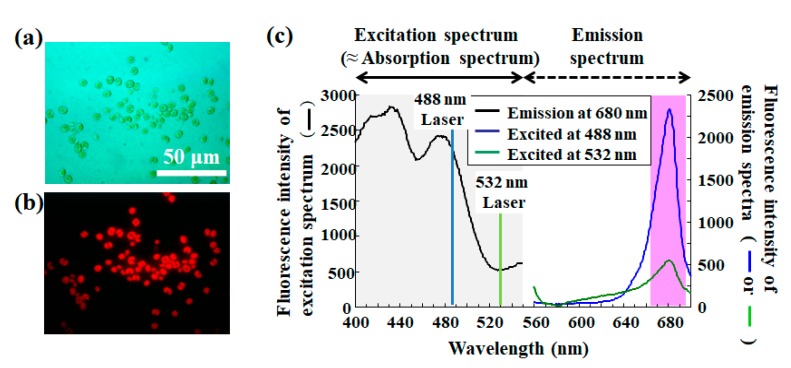
Detection of microalgae using chlorophyll autofluorescence. (**a**) Bright field image of *Chlorella*-like and symbiotic microalgae isolated from ciliate *Paramecium bursaria*, and (**b**) the corresponding fluorescence image of the algae irradiated by the mercury lamp of fluorescence microscopy. (**c**) Fluorescence properties of symbiotic algae isolated from *P. bursaria*. The graph shows excitation (black curve) and emission (blue curve and green curve, respectively) spectra of algae suspended in CA medium for microalgae: the fluorescence intensities at 680 nm for the excitation spectrum; the fluorescence intensities excited at 488 nm (blue curve) or 532 nm (green curve), respectively, for the emission spectra. Here, two vertical lines signifying 488 nm (blue) and 532 nm (green) show each excitation light source for the emission spectra. Here, parts (panels (a,b [19], c [18]) were referred from literature and modified.

Chlorophyll fluorescence is a noninvasive phenomenon for analyzing photosynthetic energy conversion of microalgae as well as cyanobacteria, phytoplankton, and higher plants [31,32]. As Figure 2 shows, chlorophyll molecules excited by an appropriate excitation light emits red florescence (Figure 3a,b). Even if a light is not most suitable for the excitation of chlorophyll, red fluorescence at approximately 680 nm is reproducibly emitted when excitation of chlorophyll occurs due to a wide range of the absorbance (excitation) wavelength (Figure 3c). Therefore, chlorophyll fluorescence allows the extraction of information regarding photosynthetic organisms from heterogeneous populations. 

**Figure 4:**
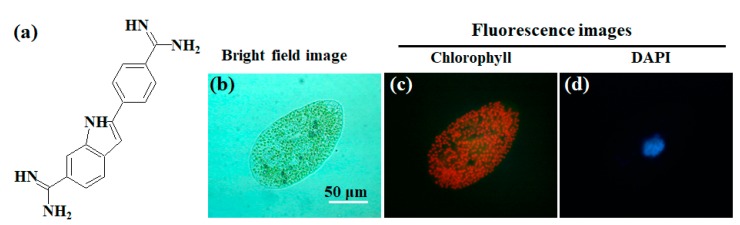
Permeability differences between a host *P. bursaria* and the symbiotic microalgae to 4′,6-diamidino-2-phenylindole (DAPI). (**a**) Molecular structure of DAPI. (**b**) A bright field image of *P. bursaria* treated with DAPI. The panels (**c**,**d**) are corresponding fluorescence images. Here, photographs in panels (b–d) were referred from literature [11].

Evidently, fluorescent analysis makes it possible to evaluate cellular and metabolic parameters using commercially available fluorescently labeled markers, such as 4′,6-diamidino-2-phenylindole (DAPI) for cell cycle analysis and BODIPY (4,4-difluoro-1,3,5,7,8-pentamethly 4-bora-3a,4a-diaza-s-indacene) for lipid analysis, rather than chlorophyll integrity. These specific fluorescence reagents or antibodies with a fluorophore can help us evaluate several cell events such as apoptosis, stage of cell cycle, gene expression, and cell metabolism, depending upon each fluorophore’s properties. Although many kinds of microalgal species are used in industrial applications, the cell walls of algae considerably differ in their permeability to dyes from species to species [33]. To visualize DNA with DAPI (Figure 4a) in algal cells, as an example, a chemical (e.g., toluene, sodium dodecyl sulfate (SDS)) or physical (e.g., freezing, microwave) treatment of algae is often carried out prior to DNA staining, because these procedures improve the permeability of organic molecule dyes such as DAPI to algal cell walls [33]. Figure 4b–d show the permeability difference for DAPI between *Chlorella*-like symbiotic algae and the host *P. bursaria* (Figure 4b) as an experimental example. Permeability difference for DAPI between the results in the corresponding difference for staining pattern are shown as negative staining of their red fluorescing symbiotic algae and positive blue staining of the host nuclei (Figure 4c,d). Although reagent permeability to cells is also generally related to cell health and integrity, it is difficult to control, modify, and optimize protocols for all industrial microalgae from species to species. As compared to protocols assisted by some organic dyes, detection of chlorophyll fluorescence does not require staining reagents and is inexpensive at each measurement. A common protocol to detect chlorophyll and evaluate their cell health can be used since almost all algae have chloroplast(s) containing chlorophyll molecules. Analysis of chlorophyll autofluorescence with no reagents necessary and detection convenience has a competitive advantage in cost-performance and availability for use in routine work. 

### 3.2. Standard Automated Cell Counter Equipped with a Fluorescence Filter

Some commercially available automated cell counters (Tali^TM^ Image-based Cytometer from Thermofisher Scientific Inc., LUNA-FL^TM^ Automated fluorescence cell counter (Logos Biosystems Inc., Anyang-si, Gyeonggi-do, South Korea), Countess^TM^ II FL automated cell counter from Thermofisher Scientific Inc., ADAM-MC2 cell counter (Montreal Biotech Inc., Doral, QC, Canada), etc.) have also been used for evaluation of more complicated cell parameters than the scope of measurement of cell numbers and cell size. These instruments can evaluate several cellular events because these have specific fluorescence filters analogous with fluorometry, fluorescence microscopy, and flow cytometry. As compared with these conventional devices, cell counters are compactly designed (device sizes and weight are, respectively: 292.1 mm (width) × 444.5 mm (depth) × 292.1 mm (height) and 8.80 kg for Tali^TM^ Image-based Cytometer; 220 mm (width) × 210 mm (depth) × 90 mm (height) and 1.8 kg for LUNA-FL^TM^ Automated fluorescence cell counter; 228.6 mm (width) × 139.7 mm (depth) × 228.6 mm (height) and 3.63 kg for Countess^TM^ II FL automated cell counter; 276 mm (width) × 227 mm (depth) × 270 mm (height) and 7 kg for ADAM-MC2 cell counter). Just to confirm, a rough measurement principle of these cell counter devices is almost identical to a conventional analysis system, which has been composed of epifluorescence microscopy closely connected with an image recording device and their data analyzing software [34,35,36,37,38,39]. Here, features such as a space-saving device design, a stand-alone device, an automatic focusing function, and ease in handling without any mature technique notice a striking difference among their cell counter products and the conventionally microscopy-based system. Moreover, automated cell counter systems do not require things like manual transference of optical fields for multipoint measurements, which operators have carried out using the conventional microscopy-based system [35,36,37]. 

The Tali^TM^ Image-based Cytometer, for instance, has three channels for bright field, green fluorescence, and red fluorescence, respectively. The green channel contains a combination of the excitation (Ex) filter (Ex 466 nm/40 nm band pass filter) and the corresponding emission (Em) filter (Em 525 nm/50 nm). The red channel contains the Ex filter (Ex 543 nm/22 nm band pass filter) and the corresponding Em filter (Em 580 nm long pass filter). Slightly different from fluorescence filters, other systems presented above such as LUNA-FL Automated fluorescence cell counter appear to be similar to the instrument. 

**Figure 5:**
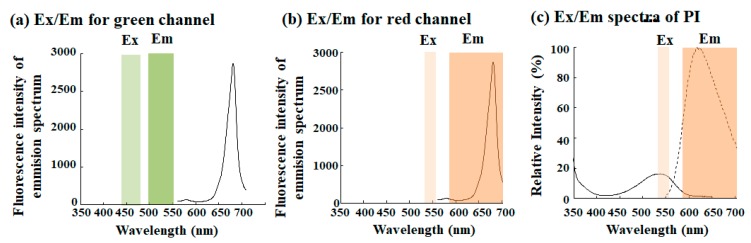
Fluorescence filters of Tali^TM^ Image-based Cytometer. Tali^TM^ Image-based Cytometer has fluorescence filters for both green (**a**) and red (**b**) channels. The panels (a,b) show each excitation (Ex) and emission (Em) filters, respectively, for the green channel and red channel. An emission spectrum of chlorophyll shown as a solid black line is of *Chlorella*-like algae isolated from *P. bursaria* in panels (a,b). In addition to Ex and Em filters for the red channel, panel (**c**) shows an excitation spectrum (black solid line) and an emission spectrum (black dotted line) of propidium iodide (PI) as an example. Here, the emission spectrum for *Chlorella*-like algae was used from Figure 3c. Both excitation and emission spectra of PI were made using online software (SpectraViewer; Thermo Fisher Scientific Inc.).

Figure 5, for instance, shows both filter sets of the Tali^TM^ Image-based Cytometer and an emission spectrum of chlorophyll autofluorescence of *Chlorella*-like microalgae excited at a 488 nm light source. Operators usually cannot change their on-board fluorescence filter sets except for the Countess^TM^ II FL automated cell counter. As those filters are optimized for frequent fluorescence proves such as green fluorescent protein (GFP), red fluorescent protein (RFP), and Alexa Fluor series reagents rather than any autofluorescence molecule in nature, some filter sets are not necessarily an appropriate choice for all autofluorescences. While the green channel is clearly mismatched for chlorophyll autofluorescence (Figure 5a), the red channel might be seemingly matched (Figure 5b). Signals from the red channel, however, might contain comprehensive information from target cells since the channel uses the long pass filter. If yellow-orange fluorescence such as propidium iodide (PI) (Figure 5c) is emitted from cells simultaneously, the red channel cannot distinguish between signals from chlorophyll and those from others. This might ruin fluorescence potential selectivity.

**Figure 6:**
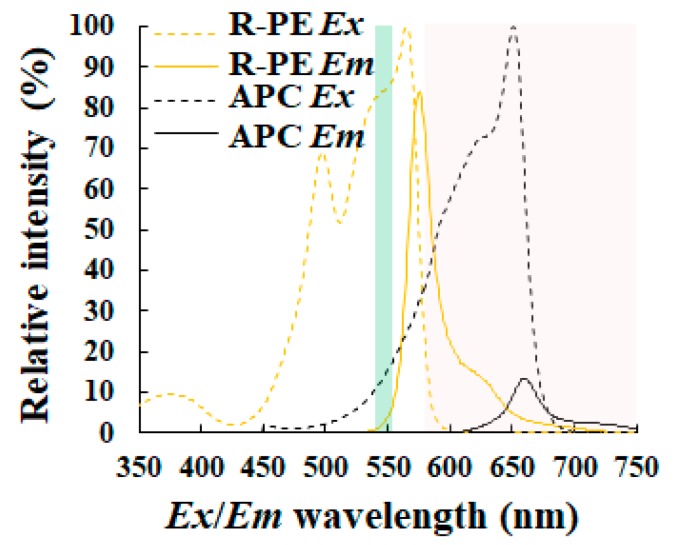
Fluorescence properties of phycobiliproteins. Both excitation (each dotted line) and fluorescence (each solid line) spectra for R-phycoerythrin (R-PE) (each orange line) and allophycocyanin (APC) (each black line), were, respectively, made using online software (SpectraViewer; Thermo Fisher Scientific Inc.) and shown. Each green and pink range shows, respectively, the excitation range and the corresponding fluorescence detection range. Here, fluorescence properties of C-phycocyanin (CPC) are not shown because they were not available using the software.

Phycobilisomes containing phycobiliproteins work as the large molecular antenna complexes for photosynthesis in cyanobacteria and red algae [40,41]. In addition to chlorophyll a, cyanobacteria have phycobiliproteins, which also emit autofluorescence [35,40,41]. R-phycoerythrin (R-PE), C-phycocyanin (CPC), and allophycocyanin (APC) are well known as phycobiliproteins [40,41]. To detect cyanobacteria, several studies have used an epifluorescence microscopy system coupled with an image recording device and their data analyzing software [34,35,36,37,38]. To excite phycobiliproteins in cyanobacteria cells, a mercury lamp and M2 green filter set (Ex 546 nm/10 nm bandpass filter, Em 580 nm long pass filter) have been used [35,36,37,38]. Figure 6, for instance, simulates fluorescence properties of phycobiliproteins such as R-PE and ACP in the case of using the above filter sets. Even if a green light is used for an excitation of chlorophyll, chlorophyll can emit weakly red fluorescence (Figure 3). Therefore, signals from the long pass fluorescence filter might contain comprehensive information. To detect and evaluate microalgal properties automatically, one absolutely needs more selective band pass filter sets than any long pass filter. 

### 3.3. Automated Cell Counter Equipped with a Fluorescence Filter to Detect Chlorophyll Fluorescence

According to the intended use, Countess^TM^ II FL automated cell counter (Thermofisher Scientific Inc., Waltham, MA, USA) unlike a conventional counter, can use several fluorescence filters (see the “EVOS Light Cube selection guide” [42]). In other words, those characteristic device designs provide an improvement to sorting out target cells from heterogeneous cell populations. As previously described in Section 3.1 and Figure 4, some organic dyes have problems with insufficient permeability of the cell membrane and cell wall of microalgae. Considering the cost-effectiveness and simplicity for use in routine work, this review specifically emphasizes detecting autogenous chlorophyll fluorescence from microalgae rather than using any fluorescent dye. 

**Figure 7:**
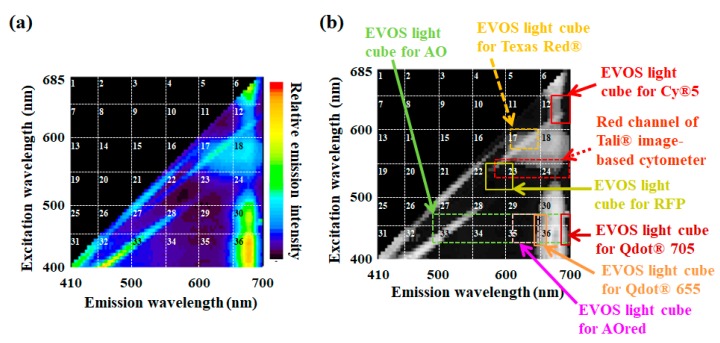
Both excitation and emission spectra of *Chlorella*-like microalgae, and selection possibility of available fluorescence filters for Countess^TM^ II FL automated cell counter (Thermo Fisher Scientific Inc.). (**a**) Three-dimensional (3D) fluorescence excitation–emission matrix spectroscopy of *Chlorella*-like microalgae was obtained using spectrofluorometry. The relatively high emission at approximately 680 nm caused by a wide range of excitation wavelengths is derived from the chlorophyll of microalgae. (**b**) The image of the 3D fluorescence fingerprint of microalgae was overlaid on both excitation and emission ranges of each commercially available fluorescence filter, targeting red fluorescence for the cell counter device (EVOS Light Cube series from Thermo Fisher Scientific Inc.). The red channel of Tali^^TM^^ Image-based Cytometer is also provided as a reference. See Table 1 for more detailed information about each filter. Here, the panel (a) was referred from literature [11].

To consider which filter is a better tool to detect chlorophyll fluorescence, three-dimensional (3D) fluorescence excitation–emission matrix spectroscopy was carried out. Figure 7a presents a graph obtained using 3D fluorescence excitation–emission matrix spectroscopy of *Chlorella*-like microalgae from *P. bursaria* [11]. As indicated in Figure 7a, the high fluorescence emissions in grid numbers 6, 12, 18, 24, 30, and 36 are algal emissions at 680 nm from chlorophyll fluorescence [11,12,18]. A difference in the excitation efficiency of chlorophyll molecules irradiated by the light of each excitation wavelength understandably lends to a greater or lesser degree of fluctuation in emission intensity. Considering the properties of chlorophyll autofluorescence from microalgae, the most suitable filter to detect chlorophyll is obviously one that can detect fluorescence in No. 36 of the grid in Figure 7a. Even with the use of the Countess^TM^ II FL automated cell counter, which has an extensive lineup for the filter from EVOS light cube series [42], the ideal filter that can excite target cells at short wavelengths of 400–450 nm and can simultaneously detect emissions at long wavelengths of 660–700 nm is rarely available. In line with chlorophyll fluorescence from microalgae, the panel in Figure 7b shows several filters for representative fluorophores with emissions at long wavelengths, like red fluorescence as an example. As a consequence of considering the better filter to detect chlorophyll fluorescence, this review offers an idea for the following measurement examples using the filter for Cy^®^5, which can detect red fluorescence in No. 12 of the grid (Figure 7a,b). 

### 3.4. Measurements of Microalgal Numbers Using the Automated Cell Counter Equipped with Fluorescence Filter for Chlorophyll Fluorescence

**Figure 8:**
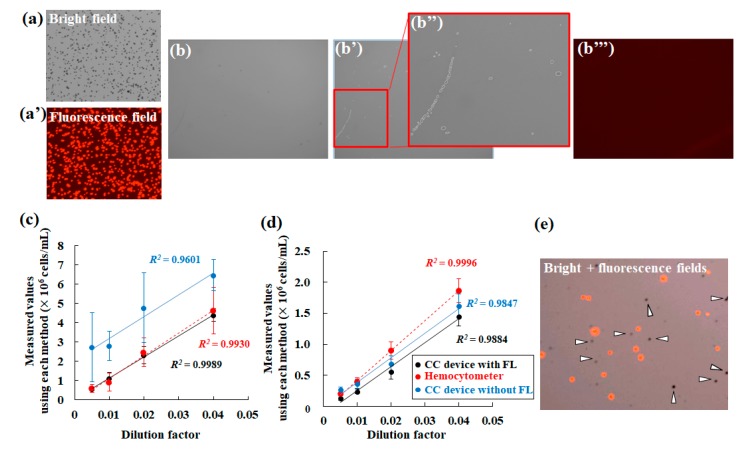
Effectiveness of fluorescence selection on detection of microalgae. (**a**,**a′**) Microalgal images in the bright field image (a) and the corresponding fluorescence image (a′) obtained from the automated cell counter. (**b**,**b′**,**b″**,**b‴**) Images of blank sample, containing distilled water only, without microalgae; the image in the bright field image (b), emphasizing detected objects with white circles by the device algorithm (b′), the magnified image (b″) of the panel (b′) and the corresponding fluorescence image of panel (b). (**c**,**d**) Comparison of each measured result of microalgal densities obtained using the cell counter device with those obtained using hemocytometry, for *Parachlorella kessleri* (c) and *Chlorella*-like alga isolated from *P. bursaria* (d). In these panels (c,d), measured values obtained using the cell counter with (black solid line; CC device with FL) or without the fluorescence filter (blue solid line; CC device without FL), and those using the hemocytometer (red dotted line) are respectively shown. (**e**) This is a measurement example of results shown in the panel (c). To explain the result in a clear depiction, the merged image of microalgae in a bright field is shown with the corresponding fluorescence image. With no fluorescence function, both non-fluorescing grimy stains and non-cell debris on the counting glass plate (white arrowheads) might cause false recognition. Here, these photographs and graphs in the panels (a–e) were referred from the literature [11] and modified.

This review introduces a method for easy and rapid evaluation of algae number and cell status using the automated cell counter (Countess^TM^ II FL cell counter) equipped with a fluorescence filter (Cy^®^5) for chlorophyll fluorescence. Just to confirm, considering both the principle of hemocytometry and the chamber volume, extremely small cells such as cyanobacteria less than 1 μm in diameter and filamentous cell population are exempt from this measurement object. This review mainly focuses on spherical microalgae larger than 1 μm in diameter because many of the microalgal species used in algal culture research and development are spherical in shape [20]. Although the microalgae tested were smaller than the recommended cell size for the device, it was still detectable (Figure 8a,a′). A blank sample test practically certifies the necessity of selectivity using fluorescent detection for chlorophyll. In practice, even if measuring a blank sample composed of distilled water (Figure 8b), an algorithm of the device to recognize cells detected unrecognizable grime on the counting chamber in bright field, in response to a very low threshold value for microalgae size (Figure 8b′,b″). Contradictory to machine recognition from the bright field image, the algorithm clearly detected no object in the fluorescence image (Figure 8b‴). The false recognition sometimes affected measurement results from the device (Figure 8c–e). Although a result from the device with fluorescence detection was almost identical to that from hemocytometry in Figure 8c, the result with fluorescence detection differed from that without fluorescence detection considerably. However, Figure 8d shows that all results were numerically approximate. The mismatch between results with and without fluorescence detection (Figure 8c) might be for precisely this reason: several particles such as bubbles and other debris along with microalgae were counted non-specifically without fluorescence detection (Figure 8e).

**Figure 9:**
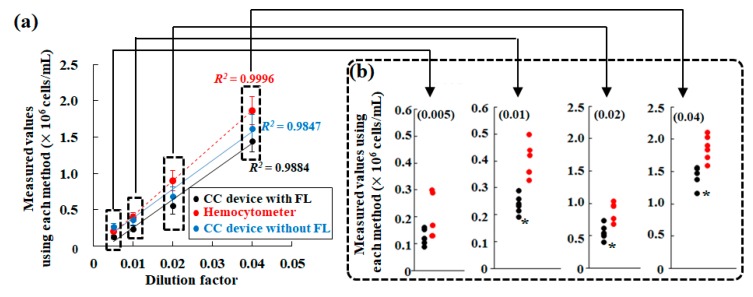
Functional and accuracy equality of the automated cell counter device with manually hemocytometry. (**a**) This panel is identical to the panel (d) shown in Figure 8. Values in this panel were used for Smirnov–Grubbs outlier testing. (**b**) To evaluate whether each value using the cell counter device (black circles) is included in the variation from hemocytometry (red circles), values from the cell counter with fluorescence function were subjected to Smirnov–Grubbs outlier testing. Here, a number noted in each bracket shows each dilution factor pointed out in the panel (a). Note that an asterisk in panel (b) denotes an outlier value to the variation from hemocytometry. Here, the panel (a) was referred from literature [11] and modified.

Takahashi [11] statistically evaluated whether measured values using the cell counter device are acceptable as an alternative from hemocytometry using a Smirnov–Grubbs outlier test. Here, the panel Figure 8d is used as an analytical example (Figure 9a). Almost every value from the automated cell counter device fell within the variation of data range using hemocytometry. Only a few values of outliers (asterisks in Figure 9b) to the variation from hemocytometry were detected. This demonstrates that an evaluation in algal cell number using the device with the fluorescence filter is statistically comparable to that of hemocytometry.

This cell counter device essentially uses the same principle of hemocytometry. Therefore, to eliminate overlap with each other, suspended cells are dispersed one by one in a tiny space (100 μm depth). Excess algal density (>approximately 10^7^ cells/mL), however, causes the overlap of cells [11], resulting in thereby-obtained inaccurate values of an algal number. Considering the overlapping of cells, cell density of ca. 10^5^–10^6^ cells/mL might be a recommended condition for the device to detect cells precisely and analogously with standard hemocytometry. 

### 3.5. Evaluation of Microalgal Size and their Status Using the Automated Cell Counter Equipped with the Fluorescence Filter for Chlorophyll Fluorescence

**Figure 10:**
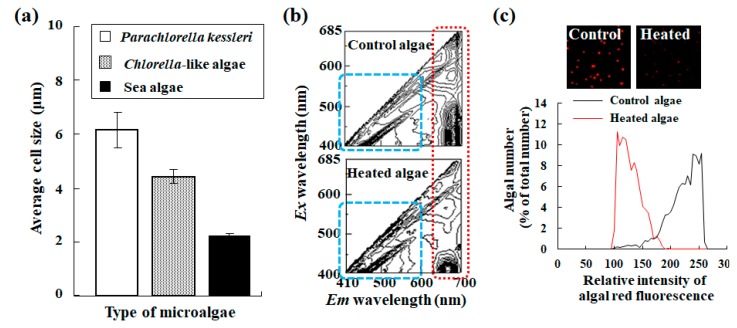
Algal parameter analysis using an automated cell counter device. (**a**) Size measurements of microalgae using the automated cell counter with a fluorescence filter for chlorophyll. Each cell size of *Parachlorella kessleri*, *Chlorella*-like symbiotic alga isolated from *P. bursaria* and sea algae were measured using the cell counter device. Here, the sea alga is related closely to a stramenopile alga in the class *Chrysophyceae* of the phylum *Stramenopiles* [11]. (**b**) These graphs show 3D fluorescence excitation–emission matrix spectrographs of *Chlorella*-like algae with or without heat treatment (for 10 min at 100 °C). A dashed red line area primarily shows chlorophyll fluorescence (see Figure 7). The dashed blue line primarily indicates the area of yellow fluorescence. (**c**) An algal status based on chlorophyll integrity was evaluated using the cell counter device before (black line) and after heat treatment (red line) of microalgae. The inset images are a fluorescence image of the control algae without heat treatment and that of heat treated algae. Note that results from *Chlorella*-like algae are shown as the example. Here, panels (a–c) were referred from literature [11] and modified.

Particle size (cell size) generally gives information about key species identification, transition of cell cycle, and foreign matter inclusion. Whereas FCM receives information of cell size from an intensity of forward-scattered light indirectly, an image based analysis such as this cell counter device can capture that from the image directly. Needing assistance in discriminating target cell population from other particles, the cell counter device can provide information about cell size. In spite of a smaller algal cell than the recommended cell size (10 μm in diameter), this device system can evaluate not only cell number but also algal cell size (Figure 10a). In a comparison of values when using the cell counter device with those using manual image analysis software, it demonstrated that data in the average size using the cell counter device were within an allowable range. Thus, their values are included in variation of diameters using manual image analysis such as ImageJ [11].

Considering the sensitivity of microalgae to changes in the culture environment [13], their quality assessment is important. Therefore, monitoring changes in chlorophyll helps us in detecting minute alterations in microalgae [11,12,14,18,19,43]. To put it briefly, heat treatment of microalgae for 10 min at 100 °C, as an example, weakens red fluorescence emission from chlorophyll (red area in Figure 10b) [11] and its treatment increases yellow fluorescence emission in reverse (blue area in Figure 10b) [12,14,18,19,31]. Figure 10c shows whether the cell counter device can detect and evaluate an algal state change associated with heat treatment. As is clear from the change in each algal appearance (inset images in Figure 10c), heat treatment of microalgae drastically reduced their red fluorescence. Histogram analysis of data obtained using the cell counter device provides a clear and quantitative indication of algal status with or without heat treatment (Figure 10c). In addition to cell number and cell size in an algal culture, the results demonstrate that the cell counter device can sensitively evaluate the algal status. As described earlier, algal status changes depending on culture conditions such as nitrogen starvation and high light stress. Even if in such a case, the cell counter has excellent flexibility for obtaining precise data. As an analogy with other devices and methods, one can set up each preset threshold level for each test alga species before an experiment. Microalgae showing out-of-range values, however, are also measured and recorded automatically. Therefore, one can change the threshold level as necessary during cell counting estimation. These features help us check reproducibility of the measurements and calibration accuracy. 

### 3.6. Remaining Issues about Application of the Automated Cell Counter to Microalgal Researches

Some kinds of spherically unicellular algae including *Dunaliella salina*, *Chlorella* sp., *Hematococcus pluvialis,* and *Coelastrella striolata var. multistriata* have already been used as valuable microalgae for industrial use for production of canthaxanthin, astaxanthin, α-carotene and β-carotene, lutein, neoxanthin, violaxanthin, zeaxanthin, and others [44,45]. Cyanobacteria and filamentous microalgae such as *Spirulina platensis*, however, are also important biomass in both ecological roles and industrial uses. Moreover, although not small, there are amorphous or filamentous microalgae such as *Botryococcus branuii* [46] to produce biofuels and *Arthrospira* sp. [45] to produce astaxanthin, zeaxanthin, lutein, and others. 

**Figure 11:**
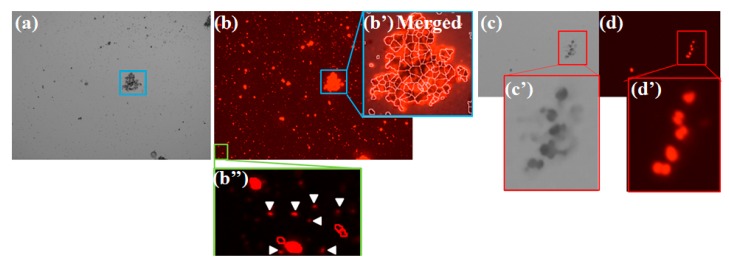
Detection ability of photosynthetic microbes other than unicellular and sphere algae using the cell counter device. A pond sample from environment (see [11] for the detail) is shown in panel (**a**) and the corresponding fluorescence image (**b**). The sample contained some cell aggregates and extremely small cells. Panels (**b′**,**b″**) respectively portray magnified images of the corresponding panel (b). Panel (b′) depicts a merged image of the bright image with the corresponding fluorescence image. A river sample (see [11]) is also shown in panel (**c**) and the corresponding fluorescence image (**d**). Panels (**c′**,**d′**) respectively depict magnified images of panels (c,d). Here, all panels (a–d) were referred from literature [11] and modified.

Here, detection capability of amorphous or small phytoplankton using the cell counter device was also tested [11]. As a result, the device with chlorophyll fluorescence detection apparently estimated each individual cell even if microalgae had aggregated [11] (Figure 11a–d). Although the device clearly seems to detect an individual cell in some adjacent cells (Figure 11c,d), it is unclear whether the device can precisely detect an individual alga in a highly aggregated cell population (Figure 11b). In practice, even if using hemocytometry and fluorescence microscopy, accurate cell counts of samples containing clumpy and aggregated cells are difficult. Although some cell aggregates might be fundamentally dispersed according to the principle of hemocytometry, other aggregates might remain. To eliminate inaccurate determination of cell numbers, physical dispersion pretreatment such as pipetting might be useful. 

In contrast to cell aggregates, smaller cells than 1 μm in diameter did not estimate despite their clear red fluorescence being captured (Figure 11b″). It is a simple reason that the device cannot focus on those cells because of very small microalgae [11]. It might be improvable if a cell counting chamber for small cells such as a bacterial counter is prepared. 

Here, although there are some challenges to do, this review supplements some points about the application of the device to cyanobacterial samples for future research and development. As mentioned previously, cyanobacteria have both chlorophyll a molecules and phycobiliproteins [40,41]. The automated cell counter device can evaluate two types of fluorophores simultaneously. Moreover, some phycobiliproteins such as R-phycoerythrin, C-phycocyanin, and allophycocyanin, have been frequently used for immunoassay techniques [47]. Therefore, simultaneous detection of both chlorophyll and phycobiliproteins might work as a non-destructive, selective and powerful tool to evaluate cyanobacteria. 

## 4. Conclusions

In view of growing applications with microalgae, the maintenance of their quality in culture is routine, yet critically important work. Considering the sensitivity of microalgae to environmental changes and culture condition, the assessment of their quality is a fundamental technology in algal application. 

Listing the several features of standard methods for microalgae, this review focused on chlorophyll autofluorescence emitted from microalgae for easy and rapid evaluation of microalgae. The automated cell counter device with a fluorescence filter for chlorophyll can precisely distinguish a unicellular alga from other debris and non-fluorescent particles. Unlike hemocytometry, this device is time-saving. In addition, it can offer technology helpful in assessing not only the number of algae but also both algal size and cell status. The cell counter device without user bias allows easy routine management of algal cultures, rapid monitoring of aberrant algae, and good quality results of microalgal products. An automated cell counter device, which has features such as a space-saving device design, a stand-alone device and ease in handling, might be useful for an establishment of an automated process on an industrial production. Altogether, the cell counter device with the filter for chlorophyll fluorescence is a potentially powerful tool that can contribute to the development of algal applications and the opening up of more markets in the microalgal industry. 

## Figures and Tables

**Table 1:** Characteristic features of each fluorescence filter from the EVOS Light Cube series [42].

Filter	Wavelength Region of Excitation (nm)	Wavelength Region of Emission (nm)
EVOS light cube for AO (Acridine Orange)	419–465	488-longer wavelength than 488 nm ^1^
EVOS light cube for AOred	419–465	612–644
EVOS light cube for RFP	511–551	573–613
EVOS light cube for Texas Red^®^	570.5–599.5	604–644
EVOS light cube for Cy^®^5	608–648	672–712
EVOS light cube for Qdot^®^ 655	422.5–467.5	647.5–662.5
EVOS light cube for Qdot^®^ 705	422.5–467.5	685–725
Red channel of Tali^TM^ Image-based Cytometer	521–565	580-longer wavelength than 580 nm ^1^

^1^: Long pass filter.
